# Depression and quality of life in old age: a closer look

**DOI:** 10.1007/s10433-020-00573-8

**Published:** 2020-05-25

**Authors:** Felix S. Hussenoeder, Doreen Jentzsch, Herbert Matschinger, Andreas Hinz, Reinhold Kilian, Steffi G. Riedel-Heller, Ines Conrad

**Affiliations:** 1 Institute of Social Medicine, Occupational Health and Public Health, University of Leipzig, Ph.-Rosenthal-Str. 55, 04103 Leipzig, Germany; 2 Department of Medical Psychology and Medical Sociology, University of Leipzig, Leipzig, Germany; 3 Department of Psychiatry and Psychotherapy II, Section of Health Economics and Mental Health Services Research, Ulm University, Ulm, Germany

**Keywords:** Depression, Quality of life, WHOQOL-BREF, WHOQOL-OLD, Older age

## Abstract

Depressive disorders are among the most widespread mental disorders in old age, with negative consequences for quality of life (QOL). Understanding QOL as a multidimensional construct, in this article we have a closer look on what specific aspects are affected by depression. We used a representative sample of the German population (*n* = 805) and one of individuals diagnosed with depression (*n* = 106) to compare QOL using the WHOQOL-BREF and the WHOQOL-OLD. Multivariate analysis showed that individuals diagnosed with depression exhibited lower QOL with regard to WHOQOL-BREF-dimensions physical health, psychological, social relationships and global QOL and with regard to WHOQOL-OLD-facets sensory abilities, past, present, and future activities and social participation. In addition, in the regression analysis, there were no significant differences between individuals with and without depression with regard to environment (WHOQOL-BREF), autonomy, death and dying, intimacy and overall (WHOQOL-OLD). Associations between depression and QOL in older age are selective in terms of which aspects of QOL are affected. From a methodological perspective, a multidimensional approach to QOL is recommended. From a clinical perspective, our research highlights those areas of QOL that are relevant for health professionals working with older people and that could be the focus of interventions.

## Introduction

Depression is a common mental disorder and a leading cause of disability worldwide (WHO 2020). Those affected by it experience depressed mood as well as reduced interest, enjoyment, energy and activity for more than two weeks. Often depression is accompanied by additional symptoms like changes in sleep and appetite, anxiety symptoms, feelings of guilt and low self-worth or poor concentration. Depressive disorders are among the most widespread mental disorders in old age, with a prevalence of 7.2% (95% CI 4.4–10.6%) for major depression, and of 17.1% (9.7–26.1%) for depressive disorders below clinical depression criteria, among those aged 75 and above according to a meta-analysis (Luppa et al. 2012). In addition, the European MentDis_ICF65+ study found 12-month prevalence rates of 11.6% (9.5–13.6%) for major depressive episodes, 2.9% (2.3–3.5%) for dysthymia, and 2.5% (1.3– 3.7%) for any bipolar disorder in an age-stratified, random sample of 3142 men and women between 65 and 84 years in Western countries (Andreas et al. 2017).

Quality of life (QOL) can be defined as an “individuals’ perception of their position in life in the context of the culture and value systems in which they live and in relation to their goals, expectations, standards and concerns” (THE WHOQOL GROUP 1995). Sivertsen et al. (2015) found a clear association between the affective disorder and QOL in older persons. While their review is explicitly focused on older individuals and many researchers agree on a multidimensional concept of QOL including physical, psychological, social and daily life aspects (Cao et al. 2016; Chang et al. 2016; Margis et al. 2010), only four out of 74 reviewed studies used multidimensional measurement that was specifically adapted to older populations, i.e., the WHOQOL-OLD (Chachamovich et al. 2008; Dragomirecká et al. 2008; Halvorsrud et al. 2010; Margis et al. 2010). All of them used the Geriatric Depression Screening Scale (GDS) (Sheikh and Yesavage 1986; Yesavage and Sheikh 1986), i.e., no clinical diagnosis, for the assessment of depression. The only study that included German participants (Chachamovich et al. 2008) did not present results specifically for the German population. In addition, also more recent studies lack depression diagnosis by health professionals and are conducted in a Brazilian setting (Bottan et al. 2014; Campos et al. 2014).

While the connection between depression and QOL seems quite straightforward, research suggests that not all QOL facets are affected in the same way (Monteiro et al. 2016; Skevington and McCrate 2012). In this article, we are especially interested in how far depression is associated with specific facets of QOL in older people. We used the multidimensional, German version of the WHOQOL-BREF (Conrad et al. 2016; THE WHOQOL GROUP 1998) to address the following dimensions: (1) physical status and in how far it allows a person to participate in daily activities (*Physical Health*), (2) psychological status, e.g., the ability to experience positive feelings and satisfaction and to concentrate (*Psychological*), (3) social relationships including support from friends, personal relationships and sex life (*Social Relationships*), (4) quality of one’s environment, e.g., related to living and financial conditions and availability of health services and means of transportation (*Environment*), (5) *Global QOL*.

In addition, since our interest is in the oldest old, we assessed QOL with the WHOQOL-OLD, an instrument that specifically addresses domains that are relevant for individuals older than 60 years (Conrad et al. 2016). As Power et al. (2005) point out questionnaires that have been developed with younger populations may not exhibit the same validity when they are applied to older populations, and there may be facets of QOL that are more relevant to older individuals, e.g., related to sensory problems and communication. The instrument comprises six facets: (5) sensory impairments and in how far they affect daily life as well as the ability to communicate with others (*Sensory Abilities*); (6) the amount of autonomy, independent decision taking and ability to influence one`s future (*Autonomy*); (7) received appreciation and felt satisfaction for accomplishments in life as well as a general future outlook (*Past, Present and Future Activities*), (8) level of activity and possibilities to participate (*Social Participation*), (9) fears and attitudes related to death and dying (*Death and Dying*), (10) possibilities to experience love and affection (*Intimacy*).

We want to fill a gap in the literature by applying multidimensional age-specific measurement to a German sample and by using clinical criteria for depression diagnosis.

## Methods

### Study design and sample

We compared a representative sample of the German population with people diagnosed with depression. For the representative group, a face-to-face survey of individuals 60 years and older was conducted in Germany in 2012 (*n* = 1031). The sample, which had been stratified according to age and gender, was also used for the standardization of the German version of the WHOQOL-OLD and WHOQOL-BREF (Conrad et al. 2014).

The second group (*n* = 133) was a convenience sample of patients from in- and outpatient settings diagnosed with depression according to ICD 10. Patients were recruited in two German cities, and the sample was also part of the WHOQOL standardization process. More detailed information on sampling procedures can be found elsewhere (Conrad et al. 2016).

Due to missing values and to the exclusion of participants in the general population that stated having mental health problems, the analytic sample contained 805 (general population, 52.2% female) and 106 (patients, 73.6% female) individuals. Ethical approval was obtained.

### Assessment

Age, gender, current living situation and highest educational qualification were assessed. In addition, the number of diseases, including hypertonia, increased cholesterol, varices, heart diseases, gastritis and diseases related to joints and spine, was assessed and counted (number of chronic diseases).

The ability to carry out instrumental activities of daily living (IADL) was assessed with the Lawton-and-Brody IADL-scale (Barberger-Gateau et al. 1992).

The DemTect was used in order to assess general cognitive status including memory, verbal fluency and attention (Kalbe et al. 2004). It is a screening test that includes (1) a word list, (2) a number transcoding task, (3) a word fluency task, (4) digit span reverse, (5) delayed recall of the word list. Scores between 13 and 18 indicate cognitive powers appropriate for subject’s age, scores between 9 and 12 point to mild cognitive impairment and scores between 0 and 8 could implicate dementia.

### Quality of Life

Quality of life was assessed using the German 26-item version of the WHOQOL-BREF, a short form of the WHOQOL-100 (Angermeyer et al. 2002). It contains the dimensions *Physical Health* (7 items), *Psychological* (6 items), *Social Relationships* (3 items), *Environment* (8 items) and *Global QOL* (2 items).

In addition, the WHOQOL-OLD was used, a test that had been specifically designed to assess the subjective QOL of adults over the age of 60. It includes six facets, with four items each: *Sensory Abilities*; *Autonomy*; *Past, Present and Future Activities* (assesses received appreciation and felt satisfaction for accomplishments in life as well as a general future outlook); *Social Participation*; *Fears related to Death and Dying*; *Intimacy* (Conrad et al. 2014, 2016; Power et al. 2005).

For both scales, scores can range from zero to 100, with higher scores representing better QOL.

### Statistical analyses

Stata version 15.1 was used for the statistical analysis. We compared means between general population and patients diagnosed with depression using independent t-tests. Furthermore, we used multiple linear regressions to analyze the effects of depression on the different measures of QOL controlling for age, gender, marital status, living situation, education, number of chronic diseases, daily living skills and cognitive status.

## Results

### Descriptive characteristics

Our dataset contained 911 individuals with 498 females (54.7%) of which 805 (52.2% females) were part of the general population and 106 (73.6% females) were individuals diagnosed with depression. Those diagnosed with depression were more likely to be female and to live with relatives. Furthermore, they were less likely to live with their partner and exhibited differences with regard to educational attainment. Table 1 displays the general characteristics of the study population.

**Table 1:** General characteristics of the study population

	Total group (*N* = 911)	General population (*N* = 805)	Individuals with depression (*N* = 106)
Age (Mean)	71.0 (8.0)	71.1 (8.1)	70.5 (7.1)
Gender (female)***	498 (54.7%)	420 (52.2%)	78 (73.6%)
Marital status			
Married and living together	477 (52.4%)	430 (53.4%)	47 (44.3%)
Married and living seperated/divorced	123 (13.5%)	101 (12.5%)	22 (20.8%)
Single	25 (2.7%)	24 (3.0%)	1 (0.9%)
Widowed	286 (31.4%)	250 (31.1%)	36 (34.0%)
Living situation*			
Alone	378 (41.5%)	327 (40.6%)	51 (48.1%)
With partner	507 (55.7%)	458 (56.9%)	49 (46.2%)
With relatives	26 (2.9%)	20 (2.5%)	6 (5.7%)
Education (Degree) ***			
None	39 (4.3%)	27 (3.4%)	12 (11.3%)
Secondary school 1^a^	476 (52.3%)	435 (54.0%)	41 (38.7%)
Secondary school 2^b^	301 (33.0%)	263 (32.7%)	38 (35.8%)
HEEQ^c^	95 (10.4%)	80 (9.9%)	15 (14.2%)
Number of chronic diseases (Mean)***	5.3 (3.8)	4.9 (3.6)	8.3 (4.3)
Daily living skills (IADL)^4^ (Mean)***	6.8 (1.6)	6.7 (1.6)	7.2 (1.3)
Cognitive status (DemTect)^5^ (Mean)	14.1 (3.4)	14.1 (3.5)	14.3 (2.5)

*p ≤ 0.05; +**p ≤ 0.01; ***p ≤ 0.001. Continuous variables are given as mean (standard deviation), and p-values refer to independent t-tests; categorical variables are displayed as numbers (percentages), and p-values refer to Chi ^2^ –tests

^a^Eight to nine years of school (German: Hauptschule)

^b^Ten years of school (German: Realschule, Polytechnische Oberschule, Fachhochschulreife)

^c^HEEQ = Higher education entrance qualification (German: Allgemeine oder Fachgebundene Hochschulreife/Abitur)

^4^Representative score for the German population M: 6.7 (SD: 1.7) (Conrad et al. 2016)

^5^Scores: 13–18 = age-appropriate cognitive abilities; 9–12 = mild cognitive impairment; 0–8 = suspected dementia (Kalbe et al. 2004)

### Group Comparisons: Quality of Life General Population versus Depressed

Table 2 shows QOL raw mean differences between the general population and individuals diagnosed with depression. The general population exhibits significantly higher scores on all WHOQOL-BREF-dimensions but Environment, i.e., Physical Health, Psychological, Social Relationships and Global QOL. Concerning the WHOQOL-OLD-facets, the general population shows significantly better scores on Sensory Abilities, Autonomy, Past, Present, and Future Activities, Social Participation, and Overall, but no significant differences with regard to Death and Dying and Intimacy.

**Table 2:** Differences in QOL between the general population (*n* = 805) and individuals diagnosed with depression (*n* = 106)

	General population mean (SE)	Individuals with depression mean (SE)	Significance
WHOQOL-BREF			
Physical Health	69.27 (0.73)	53.40 (1.74)	***
Psychological	72.16 (0.56)	52.44 (1.65)	***
Social relationships	68.30 (0.63)	61.11 (2.18)	**
Environment	73.82 (0.55)	70.83 (1.45)	n.s.
Global QOL	64.74 (0.67)	46.46 (2.00)	***
WHOQOL-OLD			
Sensory abilities	77.27 (0.70)	71.17 (2.15)	**
Autonomy	69.21 (0.68)	65.21 (1.59)	*
Past, Present and Future Activities	66.02 (0.58)	60.44 (1.56)	***
Social Participation	69.66 (0.71)	59.67 (1.78)	***
Death and Dying	62.78 (0.86)	59.73 (2.77)	n.s.
Intimacy	65.50 (0.76)	64.86 (2.31)	n.s.
Overall	68.41 (0.52)	63.51 (1.19)	***

*p ≤ 0.05; +**p ≤ 0.01; ***p ≤ 0.001. QOL = quality of life. WHOQOL-BREF = World Health Organization Quality of Life instrument (short form). WHOQOL-OLD = World Health Organization Quality of Life instrument (add-on for individuals over 60 years). The significance of mean differences was analyzed using independent *t* tests

### Depression as a predictor of QOL

Table 3 shows the regression analysis with depression as a predictor of WHOQOL-BREF-dimensions and control variables. Results show that individuals from the general population exhibit significantly better scores in terms of Physical Health, Psychological, Social Relationships and Global QOL. There are sporadic effects of age, gender, marital status, living situation and higher educational attainment on specific dimensions as well as negative effects of number of chronic diseases and positive effects of daily living skills and cognitive status on all dimensions of the WHOQOL-BREF.

**Table 3:** Impact of depression, sociodemographic and health variables on dimensions of the WHOQOL-BREF (unstandardized regression coefficients)

	Physical health (*N* = 911)		Psychological (*N* = 911)		Social relationships (*N* = 904)		Environment (*N* = 911)		Global QOL *(N *=* 908)*	
	*B*	95% CI	*B*	95% CI	*B*	95% CI	*B*	95% CI	*B*	95% CI
Depression	− 9.74***	− 13.23; − 6,25	− 16.56***	− 19.60; − 13.52	− 5.82**	− 9.55; − 2.08	− 1.16	− 4.21; 1.89	− 13.22***	− 16.89; − 9.55
Age	− 0.42***	− 0.58; − 0.26	− 0.14*	− 0.29; − 0.00	0.02	− 0.16; 0.19	− 0.03	− 0.17; 0.11	− 0.14	− 0.31; 0.03
Gender	− 1.80	− 4.17; 0.57	− 0.82	− 2.88; 1.24	2.96*	0.44; 5.48	− 2.06*	− 4.13; − 0.00	− 0.36	− 2.85; 2.12
Marital status										
Married +living seperated/divorced	− **0.49**	− **6.40; 5.42**	− 2.31	− 7.45; 2.84	− 6.37*	− 12.65; − 0.10	− 2.84	− 7.99; 2.32	2.49	− 3.72; 8.69
Single	− 2.42	− 10.90; 6.06	− 1.82	− 9.19; 5.56	− 8.94	− 17.94; 0.06	0.59	− 6.80; 7.98	3.50	− 5.39; 12.39
Widowed	2.42	− 3.49; 8.33	0.59	− 4.56; 5.73	− 0.81	− 7.09; 5.47	1.26	− 3.90; 6.41	4.50	− 1.71; 10.70
Living situation										
With partner	3.32	− 2.46; 9.09	3.18	− 1.85; 8.20	7.80*	1.67; 13.93	3.44	− 1.59; 8.48	6.22*	0.16; 12.28
With relatives	4.47	− 1.98; 10.91	3.09	− 2.52; 8.70	0.22	− 6.74; 7.17	3.84	− 1.78; 9.46	1.76	− 5.01; 8.52
Education										
Secondary school 1^a^	1.15	− 4.22; 6.52	2.30	− 2.37; 6.97	− 1.87	− 7.76; 4.02	2.23	− 2.45; 6.91	3.32	− 2.31; 8.95
Secondary school 2^b^	2.15	− 3.45; 7.75	1.98	− 2.89; 6.85	− 2.56	− 8.68; 3.56	2.41	− 2.48; 7.29	3.13	− 2.75; 9.00
HEEQ^c^	2.65	− 3.61; 8.91	4.01	− 1.43; 9.46	− 0.40	− 7.20; 6.39	7.09*	1.63; 12.55	5.46	− 1.12; 12.04
Chronic diseases (number)	− 2.16***	− 2.46; − 1.86	− 1.10***	− 1.36; − 0.83	− 0.61***	− 0.93; − 0.29	− 0.62***	− 0.88; − 0.36	− 1.70***	− 2.02; − 1.38
Daily living skills (IADL)	2.46***	1.70; 3.22	1.74***	1.08; 2.40	1.40***	0.58; 2.22	1.60***	0.94; 2.26	2.08***	1.29; 2.88
Cognitive status	1.25***	0.90; 1.59	1.19***	0.89; 1.48	1.08***	0.72; 1.45	1.28***	0.98; 1.58	1.04***	0.68; 1.40
Constant	72.44	55.70; 89.18	55.81	41.24; 70.38	42.60	24.67; 60.52	46.39	31.80; 60.99	46.04	28.46; 63.62
*R*^2^	0.43		0.36		0.18		0.21		0.32	

*p ≤ 0.05; +**p ≤ 0.01; ***p ≤ 0.001. WHOQOL-BREF = World Health Organization Quality of Life instrument (short form). QOL = quality of life. CI = confidence interval. Ranges: Physical Health = 7.14–100.00; Psychological Health = 0.00–100.00; Social Relationships = 0.00–100.00; Environment = 15.63–100.00; Global QOL = 0.00–100.00

^a^Eight to nine years of school (German: Hauptschule)

^b^Ten years of school (German: Realschule, Polytechnische Oberschule, Fachhochschulreife)

^**c**^HEEQ = Higher education entrance qualification (German: Allgemeine oder Fachgebundene Hochschulreife/Abitur)

Table 4 shows the regression analysis with depression as a predictor of WHOQOL-OLD-facets and control variables. Results show differences with regard to Sensory Abilities, Past, Present and Future Activities and Social Participation, a negative impact of number of chronic diseases on all facets, and a positive impact of higher educational attainment, daily living skills and cognitive status on all facets besides Death and Dying. There are sporadic associations between age, gender, marital status, living situation, educational attainment and individual facets.

**Table 4:** Impact of depression, sociodemographic and health variables on facets of the WHOQOL-OLD (unstandardized regression coefficients)

	Sensory abilities (*N* = 911)		Autonomy (*N* = 911)		Past, present, future activities (*N* = 911)		Social participation (*N* = 911)		Death and dying (*N* = 911)		Intimacy (*N* = 911)		Overall (*N* = 911)	
	B	95% CI	B	95% CI	B	95% CI	B	95% CI	B	95% CI	B	95% CI	B	95% CI
Depression	− 3.88*	− 7.53; − 0.23	− .82	− 5.49; 1.86	− 3.29*	− 6.50; − 0.08	− 7.46***	− 11.18; − 3.74	2.50	− 2.76; 7.76	0.36	− 3.84; 4.56	− 2.26	− 4.91; 0.38
Age	− 0.70***	− 0.87; − 0.53	− 0.17*	− 0.34; 0.00	0.10	− 0.05; 0.25	− 0.06	− 0.23; 0.12	− 0.08	− 0.33; 0.17	− .07	− 0.26; 0.13	− 0.16**	− 0.29;- 0.04
Gender	2.53*	0.06; 5.00	− 2.73*	− 5.22; − 0.24	0.33	− 1.85; 2.51	− 1.74	− 4.26; 0.78	− 0.70	− 4.26; 2.86	2.87*	0.02; 5.71	0.09	− 1.70;1.88
Marital status														
Married living seperated/divorced	− 3.37	− 9.55; 2.80	− 8.57**	− 14.79; − 2.36	− 3.34	− 8.78; 2.10	− 5.03	− 11.33; 1.28	6.27	− 2.63; 15.17	− 3.82	− 10.93; 3.28	− 2.98	− 7.46; 1.50
Single	2.03	− 6.83; 10.89	− 7.25	− 16.17; 1.67	− 4.28	− 12.08; 3.53	− 4.10	− 13.13; 4.94	− 0.26	− 13.03; 12.51	− 4.61	− 14.80; 5.58	− 3.08	− 9.50; 3.35
Widowed	− 1.17	− 7.35; 5.01	− 3.36	− 9.58; 2.86	− 0.26	− 5.70; 5.18	− 2.47	− 8.78; 3.83	2.86	− 6.04; 11.77	0.02	− 7.09; 7.12	− 0.73	− 5.21; 3.75
Living situation														
With partner	− 1.73	− 7.76; 4.30	− 6.01	− 12.08; 0.07	2.52	− 2.79; 7.83	0.90	− 5.26; 7.05	4.71	− 3.99; 13.40	15.93***	8.99; 22.87	2.72	− 1.65; 7.09
With relatives	2.71	− 4.03; 9.45	1.55	− 5.24; 8.33	6.78*	0.85; 12.71	5.83	− 1.05; 12.70	12.25*	2.55; 21.96	13.58***	5.83; 21.33	7.12**	2.23; 12.00
Education														
Secondary School 1^a^	4.11	− 1.50; 9.72	7.67**	2.03; 13.32	1.52	− 3.43; 6.45	4.15	− 1.57; 9.87	− 6.19	− 14.28; 1.89	3.41	− 3.04; 9.86	2.44	− 1.62; 6.51
Secondary School 2^b^	4.07	− 1.79; 9.92	7.26*	1.37; 13.15	2.64	− 2.52; 7.79	3.87	− .10; 9.84	− 7.33	− 15.77; 1.10	3.39	− 3.34; 10.12	2.32	− 1.93; 6.56
HEEQ^c^	6.58*	0.04; 13.12	9.95**	3.37; 16.54	7.25*	1.49; 13.01	6.76*	0.09; 13.44	− 7.38	− 16.80; 2.05	8.61*	1.08; 16.13	5.30*	0.56; 10.04
Chronic diseases (number)	− 1.18***	− 1.50; − 0.87	− 0.87***	− 1.18; − 0.55	− 0.95***	− 1.23; − 0.67	− 1.17***	− 1.49; − 0.85	− 1.73***	− 2.18; − 1.27	− 0.38*	− 0.74; − 0.01	− 1.05***	− 1.27; − 0.82
Daily living skills (IADL)	1.67***	0.88; 2.47	2.77***	1.97; 3.57	1.44***	0.74; 2.14	3.88***	3.07; 4.69	− 0.83	− 1.97; 0.32	1.71***	0.80; 2.62	1.77***	1.20; 2.35
Cognitive status	1.28***	0.92; 1.64	1.30***	0.94; 1.66	1.27***	0.95; 1.58	1.38***	1.01; 1.74	0.27	− 0.25; 0.79	1.11***	0.70; 1.52	1.10***	0.84; 1.36
Constant	99.67	82.17; 117.16	48.47	30.86; 66.09	32.85	17.45; 48.26	31.46	13.62; 49.31	80.79	55.58; 106.01	30.92	10.79; 51.04	54.03	41.35; 66.71
*R*^2^	0.33		0.22		0.21		0.30		0.08		0.23		0.32	

*p ≤ 0.05; +**p ≤ 0.01; ***p ≤ 0.001. WHOQOL-OLD = World Health Organization Quality of Life instrument (add-on for individuals over 60 years).CI = confidence interval. Ranges: Sensory Abilities = 0.00–100.00; Autonomy = 0.00–100.00; Past, Present, Future Activities = 18.75–100.00; Social Participation = 0.00–100.00; Death and Dying = 0.00–100.00; Intimacy = 0.00–100.00; Overall = 20.14–96.88

^a^Eight to nine years of school (German: Hauptschule)

^b^Ten years of school (German: Realschule, Polytechnische Oberschule, Fachhochschulreife)

^c^HEEQ = Higher education entrance qualification (German: Allgemeine oder Fachgebundene Hochschulreife/Abitur)

## Discussion

Our results show that there are differences between older individuals from the general population and those diagnosed with depression regarding WHOQOL-BREF-dimensions Physical Health, Psychological, Social Relationships, and Global QOL, and WHOQOL-OLD-facets Sensory Abilities, Autonomy, Past, Present and Future Activities, Social Participation and Overall. Regression analysis showed that individuals diagnosed with depression exhibited lower QOL with regard to WHOQOL-BREF-dimensions Physical Health, Psychological, Social Relationships and Global QOL and with regard to WHOQOL-OLD-facets Sensory Abilities, Past, Present and Future Activities and Social Participation. In all cases, the general population exhibited better QOL, a result that matches with other studies that show negative effects of depression on different aspects of QOL (Cao et al. 2016; Chang et al. 2016; Diefenbach et al. 2012; Helvik et al. 2016; Ho et al. 2014) and the fact that QOL can improve after the remission of depression (Helvik et al. 2016). In addition, number of chronic diseases, daily living skills and cognitive status had significant impact on QOL.

Similar to Cao et al. (Cao et al. 2016), we found that older individuals diagnosed with depression score significantly lower on Physical Health and Psychological. These results fit with the elevated prevalence of somatization in older individuals together with the ability to acknowledge psychological distress described in a review on the topic (Sheehan and Banerjee 1999).

Individuals diagnosed with depression exhibited reduced QOL with regard to Social Relationships (WHOQOL-BREF) that matches with the observation that depression is connected to social withdrawal and isolation, especially in an older population (Alpass and Neville 2003). While social isolation to some extent can be seen as a symptom of depression, there is also research suggesting that social isolation and loss are risk factors for depression (Choi et al. 2015; Courtin and Knapp 2017; Stein et al. 2019). This is especially problematic since older individuals, e.g., due to leaving the workforce, death of loved ones or mobility limitations, are more likely than younger individuals to experience loneliness and isolation. The withdrawal from social and fulfilling activities is also reflected in lower Social Participation scores (WHOQOL-OLD) of individuals with depression. Compared to the previously discussed WHOQOL-BREF-dimension, Social Participation is less focused on relationships and more on participation in (social) activities. Given the central role that social interaction plays with regard to depression and quality of life, but also for dementia and age-related cognitive decline (Hussenoeder and Riedel-Heller 2018), health interventions targeting older individuals diagnosed with depression should incorporate social elements, e.g., group activities and events.

Individuals diagnosed with depression exhibited significantly lower QOL regarding Sensory Abilities which fits well with current research trying to establish a causal link between sensory loss and depression (Cosh et al. 2018; Han et al. 2018; Hsu et al. 2016). It also highlights the importance of screening for mental health problems when older individuals consult their physician about changes in sensory abilities. In addition, the increased self-focus associated with depressive disorders (Grimm et al. 2009; Northoff 2007) as well as other problematic cognitive processes related to depression, including increased elaboration of and attention bias toward negative information (Kircanski et al. 2012; LeMoult and Gotlib 2018), may to some extent explain group differences in Sensory Abilities as well as Past, Present, and Future Activities. Surprisingly, in the regression analysis there are no significant differences between the general population and individuals diagnosed with depression with regard to Autonomy, Death and Dying, and Intimacy. Hence, depression seems to have no impact on these dimensions of QOL. Future research may benefit from analyzing the effects of different stages and conditions of old age since, for example, autonomy may become more relevant with higher age.

From a methodological perspective, our work clearly shows that a multidimensional approach to QOL in older individuals is useful since not all facets/dimensions are affected in the same way.

## Limitations

While this study has several advantages, e.g., the comprehensive assessment of depression and QOL, there are also certain limitations. For example, further research would benefit from including information on depression history and applying a longitudinal design. In addition, there is a certain overlap between the concepts of depression and QOL that could bias results, and future research may benefit from a larger sample of individuals diagnosed with depression.
